# A mesophilic anaerobic digester for treating food waste: process stability and microbial community analysis using pyrosequencing

**DOI:** 10.1186/s12934-016-0466-y

**Published:** 2016-04-25

**Authors:** Lei Li, Qin He, Yao Ma, Xiaoming Wang, Xuya Peng

**Affiliations:** Key Laboratory of Three Gorges Reservoir Region’s Eco-Environment, Ministry of Education, Chongqing University, Chongqing, 400045 China

**Keywords:** Food waste, Anaerobic digestion, Microbial community, Process stability, OLR disturbance

## Abstract

**Background:**

Anaerobic digesters become unstable when operated at a high organi c loading rate (OLR). Investigating the microbial community response to OLR disturbance is helpful for achieving efficient and stable process operation. However, previous studies have only focused on community succession during different process stages. How does community succession influence process stability? Is this kind of succession resilient? Are any key microbial indicator closely related to process stability? Such relationships between microbial communities and process stability are poorly understood.

**Results:**

In this study, a mesophilic anaerobic digester for treating food waste (FW) was operated to study the microbial diversity and dynamicity due to OLR disturbance. Overloading resulted in proliferation of acidogenic bacteria, and the resulting high volatile fatty acid (VFA) yield triggered an abundance of acetogenic bacteria. However, the abundance and metabolic efficiency of hydrogenotrophic methanogens decreased after disturbance, and as a consequence, methanogens and acetogenic bacteria could not efficiently complete the syntrophy. This stress induced the proliferation of homoacetogens as alternative hydrogenotrophs for converting excessive H_2_ to acetate. However, the susceptible *Methanothrix* species also failed to degrade the excessive acetate. This metabolic imbalance finally led to process deterioration. After process recovery, the digester gradually returned to its original operational conditions, reached close to its original performance, and the microbial community profile achieved a new steady-state. Interestingly, the abundance of *Syntrophomonas* and *Treponema* increased during the deteriorative stage and rebounded after disturbance, suggesting they were resilient groups.

**Conclusions:**

Acidogenic bacteria showed high functional redundancy, rapidly adapted to the increased OLR, and shaped new microbial community profiles. The genera *Syntrophomonas* and *Treponema* were resilient groups. This observation provides insight into the key microbial indicator that are closely related to process stability. Moreover, the succession of methanogens during the disturbance phase was unsuitable for the metabolic function needed at high OLR. This contradiction resulted in process deterioration. Thus, methanogenesis is the main step that interferes with the stable operation of digesters at high OLR. Further studies on identifying and breeding high-efficiency methanogens may be helpful for breaking the technical bottleneck of process instability and achieving stable operation under high OLR.

## Background

Anaerobic digestion (AD) of organic waste is considered to be a sustainable waste treatment practice, as it reclaims potential energy from waste in the form of biogas and provides a route by which nutrients can be recycled back to land [1, 2]. Food waste (FW) is an organic waste with high bio-methane potential. Nowadays, FW is generated at an ever-increasing rate in China, and improper disposal and treatment can cause serious environmental problems and public health risks. Therefore, AD has rapidly become a widespread practice [3, 4]. Among the many (83) methods for FW disposal implemented in China, AD is dominant (>90 %) (http://digitalpaper.stdaily.com/http_www.kjrb.com/kjrb/html/2015-02/03/content_292023.htm?div=-1). However, “process stability” is a key factor related to the success of FW digesters’ operation. Operating digesters always suffer from instabilities such as inhibition, acidification, and foaming, especially at high organic loading rates (OLR) [3–5]. Such process instabilities are generally associated with the characteristics and complexities of the microbial communities involved in the AD process. During AD, organic matter is converted into biogas through four consecutive steps including hydrolysis, acidogenesis, acetogenesis, and methanogenesis [1, 2]. Each stage involves different functional microbial groups, which are highly interactive and have different growth rates, physiologies, and nutritional needs. Thus, the metabolic balance within these distinct microbial groups is fragile, and imbalance in any single degradation step will disturb the whole process [5, 6].

Consequently, investigating the composition and behavior of microbial communities in digesters is helpful for efficient and stable process operation. Researchers have explored microbial community structure in many full-scale anaerobic digesters to facilitate digester management [6–9]. Some studies have even linked OLR disturbance with microbial communities. For example, Sundberg et al. and Belostotskiy et al. studied community shifts in digesters with different OLRs under steady-state conditions [5, 10]. A few authors have taken the deteriorative phase into consideration. For instance, Polag et al. studied the dynamics of microbial composition and population in digester operated under highly variable loading conditions including under and overfeeding conditions [11]. Razaviarani and Buchanan investigated the link between reactor performance and microbial communities under steady-state and overloaded conditions in the co-digestion of municipal wastewater sludge with restaurant grease waste [12]. However, current literature has only highlighted community succession during different process states, and little information is available on the relationship between this kind of succession and process deterioration. In addition, is community succession reversible? Are there any key microbial indicators that can be used as potential process indicators of the actual state of AD? Such relationships between microbial communities and process stability are poorly understood. Moreover, there appears to be little information on microbial succession during the recovery phase, which is thought to be of particular significance for understanding all OLR disturbance events in a digester. Therefore, it is necessary to more precisely monitor the community dynamics during each operational phase and determine how microbial communities respond to stress.

This study introduced OLR disturbances into a mesophilic anaerobic digester treating FW to induce stable (Phase I), disturbance (Phase II), recovery (Phase III), and new stable (Phase IV) stages, during which physico-chemical analysis along with the pyrosequencing microbial technique were performed to monitor state parameters and microbial communities of each phase, respectively. The objectives were to (1) clarify how community succession influences process stability; (2) investigate whether community succession is resilient or not; and (3) provide insight into the key microbial indicator closely related to the stability of anaerobic digesters.

## Results and discussion

### Reactor performance

**Fig. 1:**
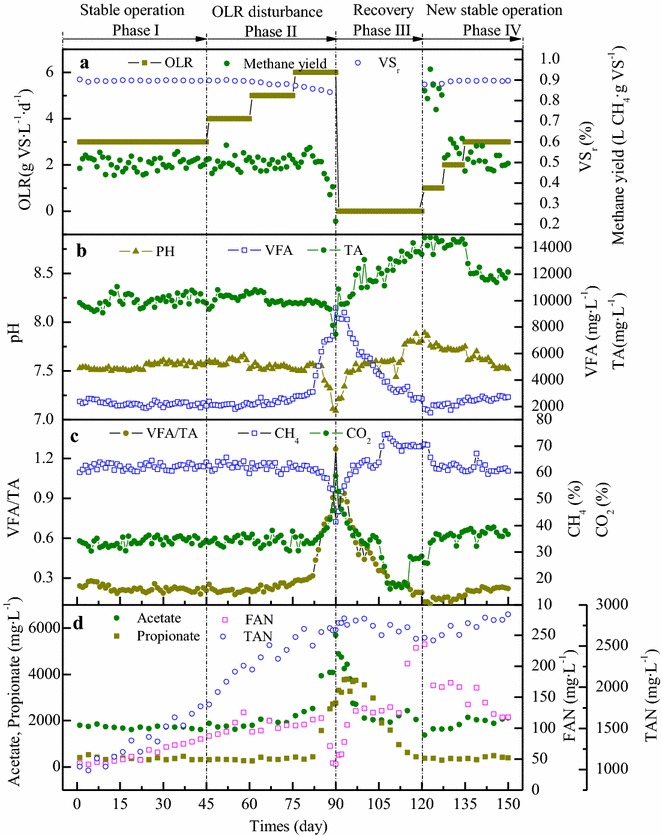
Process performance of anaerobic digester. Evolution of OLR, VS_r_, methane yield (**a**), pH, total VFA, TA (**b**), VFA/TA, CH_4_, CO_2_ (**c**) and acetate, propionate, FAN, TAN (**d**) in digester during the experiment

Time series of OLR, methane yield, volatile solid removal rate (VS_r_), pH, total volatile fatty acids (VFA) and alkalinity (TA), VFA/TA, CH_4_, CO_2_, acetate, propionate, total ammonia–nitrogen (TAN), and Free ammonia (FAN) are shown in Fig. 1. TAN and FAN increased continuously during Phase I; at Day 45, their concentrations were 1767 and 83 mg L^−1^, respectively. It has been reported that a FAN level of about 100 mg L^−1^ and TAN level of 3000 mg L^−1^ caused inhibition in an anaerobic digester [13]. Therefore, the effect of TAN and FAN on the methane yield was negligible during this stage. A stable methane yield and VS_r_ were obtained, and the other state parameters were all relatively constant, assuring a steady-state process during Phase I.

To induce the process of deterioration, a stepwise OLR disturbance was introduced during Phase II. As shown in Fig. 1, an increase in OLR from 3 to 4 g VS L^−1^ d^−1^ had no observable effect on the process efficiency or reactor stability. When the OLR was increased to 5 g VS L^−1^ d^−1^, a slight increase in VFA was observed, which was accompanied by a slight decrease in TA. This anomaly may have been caused by FAN inhibition, as the concentration of FAN exceeded 100 mg L^−1^ at Day 67. However, these parameters did not continue to deviate from their original levels, but achieved a new steady-state, and the process efficiency was not affected. When the OLR was further increased to 6 g VS L^−1^ d^−1^, FAN continuously increased to 114 mg L^−1^ at Day 82, then the VFA concentration rapidly increased from 3100 (Day 82) to 9443 mg L^−1^ (Day 90). Although acetate was still the dominant component of VFAs, propionate increased by 20-fold. In addition, the concentrations of butyrate and valerate also increased (data not shown). Fermentative microbial communities have much faster growth kinetics than methanogens. Under high OLR, the rapidly proliferating bacteria hydrolyzed organics to VFAs, but the slow-growing and even inhibited methanogens could not directly or indirectly degrade the generated VFAs in time, which resulted in VFA accumulation [14, 15]. The accumulated VFAs lowered the TA in the digester, and reduced the pH to suboptimal values, which further exacerbated the toxic effect on the methanogens. Eventually, all these factors resulted in reduced AD efficiency.

After OLR stress, process recovery is essential, and a drastic decrease in OLR is the most common way to achieve this [16]. Considering the severe acidification, the loading of the digester was halted during Phase III to accelerate process recovery. As shown in Fig. 1, VFAs gradually restored to their normal ranges as the recovery time increased. In contrast, the methane content not only recovered, but also reached a higher level. This may be because as the VFAs were consumed, HCO_3_^−^ that was previously combined with VFAs was released, causing the TA in the digester to increase. The increased TA increased the pH in the digester, resulting in less CO_2_ spilled from the liquid phase, and the relative content of gaseous methane increased. The higher TA concentration and pH at this stage confirmed this inference. The increase in pH shifted the balance between FAN and ammonium ions and caused a sharp increase in FAN, resulting in FAN >200 mg L^−1^ at Day 115. However, the high FAN level did not inhibit the process performance, possibly because the microbial communities were acclimated by the step-wise increased ammonia concentration. As reported by Yenigun and Demirel, ammonia inhibition to mesophilic AD with acclimated inoculum is triggered mostly at levels of 2800–6000 mg L^−1^ TAN and 337–800 mg L^−1^ FAN [17].

After 1 month of process recovery, the digester was re-fed. A transition period with low OLR was first introduced to reduce the loading impact; then the operational conditions of Phase IV were set as the same as those for Phase I. As shown in Fig. 1, the process performance of these two stages was comparable; high TAN (2810 ± 53 mg L^−1^) and FAN (134 ± 18 mg L^−1^) did not have a toxic effect on the AD process. The methane yield, VS_r_, and VFA/TA during these two stages were similar. However, the VFA and TA concentrations during Phase IV were slightly higher, possibly because of the microbial community shift.

### Pyrosequencing analysis

Pyrosequencing was performed to monitor the microbial community during each phase in the digester. The qualified nucleotide sequence reads were grouped into operational taxonomic units (OTUs) at a distance level of 3 % to estimate the phylogenetic diversities of microbial communities. Table 1 summarizes the sample information and statistical results used for each sample. As shown in Table 1, no significant changes were observed in the richness of archaea during the experiment; in contrast, the richness of bacteria slightly fluctuated, but the fluctuations appeared to be random. There were significant differences between community evenness among samples derived from different operational phases for both bacteria and archaea. The estimated Jaccard indices showed that the archaeal community was highly stable during the whole operational process. In contrast, the bacterial community was more dynamic, but their dynamics appeared to be correlated with time. Therefore, there was no clear correlation between these ecological parameters and process stability. Previous studies have tried to link these ecological parameters with process stability. For example, Carballa et al. and Werner et al. found a positive correlation between community evenness and performance of anaerobic reactors [18, 19]. Ziganshin et al. and Regueiro et al. concluded that bacterial diversity and richness are not associated with process stability, but archaeal populations are correlated with reactor performance [20, 21]. In contrast, Dearman et al. suggested that global microbial diversity is not important for developing a functionally successful anaerobic microbial community [22]. Thus, it is still controversial what level of community complexity a healthy, well-balanced, efficient microbial consortium should have for the production of biogas. Moreover, judging the process stability of a digester according to general ecological parameters is not a sophisticated method. Therefore, to clarify the relationship between process stability and microbial community, further investigations on specific community succession under different process stages are necessary.

**Table 1:** Sample information and statistical results

Samples	Reads	OTU	Richness	Gini coefficient	Jaccard similarity index (%)			
					Day 45	Day 90	Day 120	Day 150
Archaea								
Day 45	6221	35	36 (5)a	0.786 (6.5E−02)b	–	99.49	97.23	97.27
Day 90	6552	32	36 (11)a	0.767 (8.9E−02)a		–	99.60	99.47
Day 120	6879	31	32 (5)a	0.775 (8.3E−02)ab			–	99.78
Day 150	5892	33	40 (15)a	0.850 (5.2 E−02)c				–
Bacteria								
Day 45	6625	173	192 (15)a	0.901 (4.0E−02)a	–	74.14	73.40	73.41
Day 90	6614	189	212 (16)ab	0.949 (2.0 E−02)c		–	65.38	58.00
Day 120	7256	215	239 (17)b	0.956 (1.7E−02)d			–	96.03
Day 150	7789	196	238 (25)b	0.931 (2.5 E−02)b				–

Numbers in brackets stand for standard errors, and the different letters show a significantly different among samples at different operational phase (P < 0.05)

### Bacterial communities in response to OLR disturbance

**Fig. 2:**
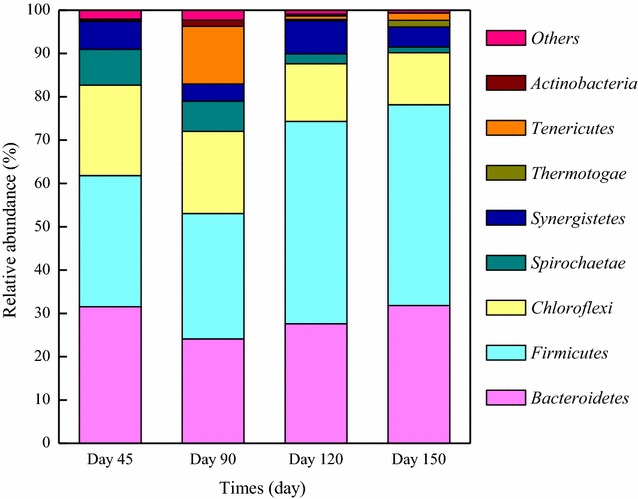
Taxonomic classification of the bacterial communities at the phylum level. Phyla making up less than 1 % of total composition in all the samples were classified as others

The bacterial sequence distributions at the phylum level are shown in Fig. 2, and Table 2 further deconstructs the bacterial sequence at the class and genus levels. The majority of sequences from Phase I were assigned to the phyla *Bacteroidetes, Firmicutes, Chloroflexi, Spirochaetae* and *Synergistete.* After the process deterioration caused by overloading, the relative abundance of the above phyla all decreased. In contrast, the abundance of *Actinobacteria* increased from 0.03 to 1.41 %, and the amount of *Tenericutes* sharply increased from 0.08 to 13.30 %. *Tenericutes*-affiliated bacteria are facultative anaerobes. Under anaerobic conditions, they produce organic acids, which can be used by acidoclastic methanogens [7]. Phylum *Actinobacteria* may also be responsible for hydrolyzing and degrading FW into VFAs, and some bacteria in *Actinobacteria* produce propionate [23, 24]. Thus, the proliferation of *Tenericutes* and *Actinobacteria* may be related to the high VFA yield during Phase II. The abundance of class *Clostridia* (phylum *Firmicutes*) also sharply increased during Phase II. Members of *Clostridia* are capable of performing diverse fermentation pathways. Apart from their role in hydrolysis and acidogenesis, they are also involved in acetogenesis and syntrophic acetate oxidation (SAO) [7, 25]. They are also efficient hydrogen producers; their proliferation suggests that excessive H_2_ was generated in the digester [26]. Once the hydrogenotrophs failed to degrade the produced H_2_ in time, the degradation of VFAs is disturbed. This may be the cause of the acid accumulation during Phase II. *Syntrophomonas* was a representative genus in class *Clostridia*. *Syntrophomonas*-related bacteria are syntrophic fatty-acid-oxidizing bacteria, which can convert various organic acids to H_2_ and acetate for subsequent hydrogenotrophic methanogenesis (HM) [27, 28]. Their proliferation during Phase II was consistent with the sharp increase in propionate and the accumulation of butyrate and valerate. The relative abundance of genus *Treponema* within phylum *Spirochaetes* also increased from 0.5 to 3.28 % during Phase II. Members of *Treponema* are likely homoacetogens, which consume H_2_ and CO_2_ to produce acetate [29]. Homoacetogenesis is typically observed under psychrophilic conditions, as homoacetogens have a better ability to adapt to low temperatures compared with hydrogenotrophic methanogens [30]. It has been reported that homoacetogenesis cannot compete with HM under mesophilic or thermophilic conditions because of its lower energy yield. However, Wang et al. observed the coexistence of *Treponema* and hydrogenotrophic methanogens in a mesophilic digester used for treating sewage sludge with H_2_ influent [29]. Siriwongrungson et al. found that homoacetogenesis can act as an alternative pathway for H_2_ consumption during thermophilic AD of butyrate under suppressed methanogenesis [30]. Thus, adverse circumstances may induce the proliferation of homoacetogens in suboptimal conditions, which may play a key role in optimizing the performance of the system. In this study, the amount of *Treponema* dramatically increased during Phase II, which may have been induced by the high H_2_ stress in the digester. Moreover, the excessive H_2_ may have been converted to methane by both direct (HM) and indirect (homoacetogenesis and acetoclastic methanogenesis (AM)) pathways.

**Table 2:** Taxonomic compositions of bacterial communities at the class and genus level

Taxonomic compositions	Relative abundance (%)			
Class/genus	Day 45	Day 90	Day 120	Day 150
*Clostridia*	*8.18*	*26.82*	*45.19*	*45.53*
*Coprothermobacter*	0.05	0.12	1.54	1.62
*Fastidiosipila*	1.69	3.69	2.83	0.62
*Gelria*	0.53	0.30	2.60	3.33
*Sedimentibacter*	0.24	1.22	0.47	0.08
*Syntrophaceticus*	0.00	0.00	2.43	1.26
*Syntrophomonas*	0.50	1.78	1.68	0.89
*Bacteroidia*	*27.58*	*23.16*	*27.43*	*31.71*
*Alkaliflexus*	0.14	1.24	4.26	0.60
*Bacteroides*	2.84	1.30	7.57	0.77
*Petrimonas*	18.85	17.54	6.92	3.50
*Proteiniphilum*	0.78	2.59	8.57	26.37
*VadinBC27_wastewater*-*sludge_group*	4.33	0.17	0.10	0.37
*Synergistia*	*6.48*	*3.98*	*7.65*	*4.57*
*Aminobacterium*	0.08	0.21	1.78	2.03
*Thermovirga*	4.82	2.24	3.25	1.13
*Spirochaetes*	*8.27*	*6.95*	*2.29*	*1.40*
*Candidatus_cloacamonas*	0.00	0.00	0.00	0.03
*Spirochaeta*	2.87	3.07	0.41	0.40
*Treponema*	0.50	3.28	1.47	0.86
*Thermotogae*	*0.11*	*0.00*	*0.30*	*1.54*
*060F05*-*B*-*SD*-*P93*	0.00	0.00	0.30	1.51
*Mollicutes*	*0.08*	*13.31*	*0.80*	*1.66*
*Acholeplasma*	0.08	13.27	0.80	1.66
*Actinobacteria*	*0.33*	*1.41*	*0.37*	*0.05*
*Actinomyces*	0.29	1.36	0.33	0.03

Only identified genera with relative abundances higher than 1.0 % in at least one sample are listed

The relative abundances of bacterial classes are in italics

During Phases III and IV, the relative abundance of class *Clostridia* continuously increased, but no VFA accumulation was observed, possibly because an efficient pathway for H_2_ consumption occurred. In contrast, the abundance of genus *Syntrophomonas* decreased during Phase III and was then restored to the same level as Phase I during Phase IV. Meanwhile, the amounts of another representative genus *Syntrophaceticus* suddenly increased during Phase III. Genus *Syntrophaceticus* has the opposite metabolic function as *Treponema*. They are syntrophic acetate oxidizing bacteria (SAOB), oxidizing acetate to CO_2_ and H_2_, which in turn can be converted into methane by hydrogenotrophic methanogens [25]. Compared to AM, the concurrent reactions of SAO and HM are less efficient for acetate degradation. However, the tolerant acetate oxidizers and hydrogenotrophic methanogens are expected to continue to function in more hostile environments [31]. As shown in Table 2, this genus was not found during Phases I or II, but a high abundance was detected during Phases III and IV, indicating the key role of SAO in acetate degradation during recovery and new stable stages. This observation reveals the inefficiency of acetoclastic methanogens, and emphasizes the importance of HM during the last two stages. Correspondingly, the abundance of genus *Treponema* decreased during Phase III and reached its original level during Phase IV. This observation indicates that the proliferation of *Treponema* is consistent with process deterioration, and it may be used as a potential warning indicator of process instability. Moreover, the relative abundance of other syntrophic bacteria also changed considerably. For example, the amounts of class *Synergistia* (phylum *Synergistetes*) increased during Phase III. This class includes numerous bacteria that can efficiently degrade complex organic materials and ferment lactic or acetic acid to H_2_ and CO_2_ [23]. Their predominance indicates that a syntrophic relationship with hydrogenotrophic methanogens occurred in the digester. The succession of class *Thermotogae* (phylum *Thermotogae*) also supported the inference. *Thermotogae* was only detected during Phase III and IV, its representative genus *060F05*-*B*-*SD*-*P93* can produce exopolysaccharides (EPS), which are used in the formation of stable cellular aggregates and facilitate interspecies H_2_ transfer [32]. These successions show the irreversible bacterial communities before and after disturbance and may also imply a shift in the methanogenesis pathway at Phase III and IV.

### Methanogen communities in response to OLR disturbance

**Fig. 3:**
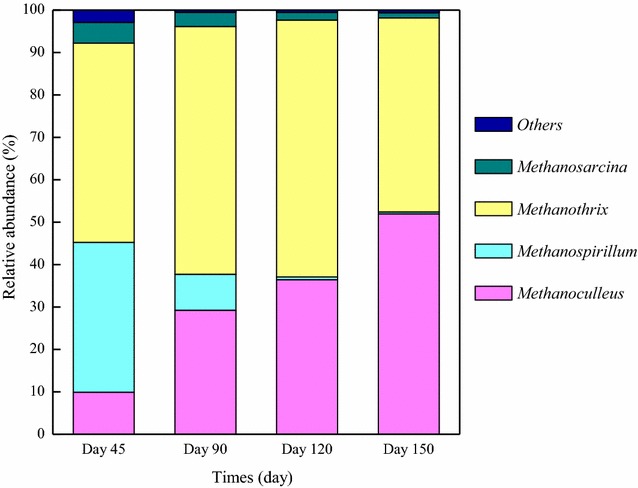
Taxonomic compositions of methanogens at the genus level. Genera making up less than 1 % of total composition in all the samples were classified as others

A shift in the metabolic pathways and metabolites of bacteria directly affects the composition and behavior of methanogens. Figure 3 shows the succession of methanogens in response to OLR disturbance at the genus level. The acetoclastic methanogen *Methanothrix* and hydrogenotrophic methanogens *Methanospirillum* and *Methanoculleus* were the dominant genera during the overall experimental period. The genus *Methanosarcina*, whose metabolic features are diverse and include both acetotrophic and hydrogenotrophic pathways, was also detected, but abundances were always low (1.24–4.90 %). Specifically, during Phase I, *Methanothrix* was the most dominant genus with an abundance of 46.97 %, followed by *Methanospirillum* (35.35 %) and *Methanoculleus* (9.89 %). The VFA accumulation and ammonia inhibition led to process deterioration during Phase II, however the relative abundance of susceptible *Methanothrix* increased to 58.47 %. This abnormal phenomenon has been discussed in our previous study [4]. Moreover, the predominant hydrogenotrophic methanogens shifted from *Methanospirillum* to *Methanoculleus*. As we know, the H_2_ affinity of *Methanoculleus* was higher than *Methanospirillum*; thus, this succession reduced the H_2_ consumption efficiency, which was not consistent with the succession of bacteria under high OLR. Generally speaking, a shift in community structure is always in the direction of the species dealing with the stress conditions and adaptations to the new environment [14]. Thus, methanogens should shift towards a genus with a higher H_2_ consumption rate such as *Methanobacterium*. However, the present study, as well as many others, identified the predominance of *Methanoculleus* under stress conditions [1, 2, 33]. Its dominance over other hydrogenotrophic methanogens may be related to its tolerance of high ammonia concentrations [1]. In addition, *Methanoculleus* species have a higher gene content compared to other genera that are involved in specific pathways and some that are directly involved in methanogenesis. More specifically, they can use different secondary alcohols as electron donors for methanogenesis [33, 34]. These features may be advantageous for the survival of *Methanoculleus sp.* in different environments and their dominant presence in digesters.

During the recovery phase (Phase III), *Methanothrix* was still the most dominant methanogen with an abundance of 60.60 %, and hydrogenotrophic methanogens were relatively low in abundance. However, it is likely that methane was mainly produced via the HM pathway, as mentioned above, the increased abundance of *Syntrophaceticus* indicates the low efficiency of acetoclastic methanogens. Moreover, with the further proliferation of syntrophic bacteria (e.g., classes *Clostridia,Synergistia* and *Thermotogae*) and the decrease in alternative hydrogenotrophs (genus *Treponema*), the process performance was restored to a normal level. The contradiction between abundance and function may be explained by microbial activity. Schauer-Gimenez et al. observed that the relative abundance of *Methanospirillum* and *Methanoculleus* was less than a quarter of that of *Methanothrix*, but the specific methanogenic activity (SMA) for H_2_ uptake was 188-fold higher than that of acetate [35]. Shigematsu et al. also found that although the proportion of *Methanothrix* in their system was as high as to 88 %, SAOB and hydrogenotrophic methanogens converted the acetate in the digester [36]. In addition, because the feed during the recovery stage was ceased, the changes in archaeal communities during Phase III might be due to the different decay rates of the archaea rather than their different growth rates. *Methanoculleus* became the most dominant methanogen during Phase IV, which may be related to the high ammonia concentration. Although the high ammonia concentration did not cause process inhibition at this stage, it changed the microbial community composition, and promoted proliferation of the tolerant *Methanoculleus.*

### Relationship between process stability and microbial community

There are three basic mechanisms for maintaining microbial community function independent of process disturbance: resistance (populations are able to withstand changes without variations in composition), resilience (populations respond to disturbances and have the ability to rebound following disturbances), and redundancy (a disturbed population can be replaced by a new group with the same function, thus a change in community composition will not affect system performance) [37]. Applying these concepts to anaerobic microbiomes, some studies have suggested that hydrolytic and acidogenic bacteria rely on functional redundancy or resistance to maintain overall function, whereas syntrophic populations tend to be more resilient [38, 39]. Some researchers have even extended these concepts to specific microorganisms. For example, Carballa et al. speculated *Methanobacteriales* as resistant, *Methanothrix* as redundant, and *Methanosarcina* and *Methanomicrobiales* as resilient and redundant [38]. Goux et al. concluded that *Bacteroidales* were resistant to high VFA and low pH [2]. The relationship between resistant and redundant populations and process stability is unintelligible, while the succession of resilient groups is closely related to the process performance. Thus resilient groups may play an important role in indicating the actual state of AD.

All three mechanisms were observed in this study. The behavior of the microbial community during the entire disturbance event is inferred and summarized as follows. The increased OLR caused the proliferation of acidogenic bacteria (phyla *Tenericutes* and *Actinobacteria*), and the resulting high VFA yield induced an increase in the abundance of syntrophic acetogenic bacteria (class *Clostridia*). However, the abundance of total hydrogenotrophic methanogens decreased, and the ammonia accumulation shifted the dominant hydrogenotrophic methanogens from *Methanospirillum* to *Methanoculleus*, which further decreased the H_2_ consumption rate. This opposite behavior resulted in the uncoupling of acetogenic bacteria and the methanogens, thus they could not effectively complete the syntrophy. This stress induced the propagation of *Treponema* as alternative hydrogenotrophs. However, *Methanothrix* species are well known as susceptible groups, and their metabolic activity may have also been affected by the high ammonia concentration. Thus, the excessive acetate was not degraded in time (Fig. 1). The mismatch between bacteria and methanogens caused the accumulation of VFAs, which lowered the pH and buffer capacity of the digester and resulted in a decrease in methane content and yield, finally resulting in process deterioration. During the recovery stage, increased metabolic activity allowed hydrogenotrophic methanogens to out-compete *Treponema*, leading to a decline in *Treponema*. Hydrogenotrophic methanogens efficiently degraded the accumulated VFAs accompanied by syntrophic partners (*Clostridia* and *Synergistia*); the depletion of VFAs resulted in a decrease in fatty-acid-oxidizing bacteria (*Syntrophomonas*). In addition, as the ammonia inhibition decreased the activity of *Methanothrix* species, the tolerant acetate oxidizers (genus *Syntrophaceticus*) proliferated. Excessive acetate was converted to methane through concurrent reactions (SAO + HM). At this time, the dominant methanogeneic pathway likely shifted from acetoclastic to hydrogenotrophic. During Phase IV, the digester was re-fed and gradually operated under the same conditions as in Phase I. Though similar process performance was observed, the microbial composition changed significantly and new steady-state microbial community profiles were shaped after the disturbance (Table 2). The overall microbial community was functionally redundant, however classes *Clostridia* and *Bacteroidia* were always the dominant groups in the digester, indicating their resistance. The genera *Treponema* and *Syntrophomonas* were sensitive to the disturbance, but rebounded afterwards, suggesting that they are potentially resilient groups. As the relative abundances of the genera *Treponema* and *Syntrophomonas* were closely related to the process stability, we infer that they may be key microbial indicators closely related to the stability of anaerobic digesters.

## Conclusions

This study investigated the microbial diversity and dynamicity during four consecutive phases (stable, deterioration, recovery, and new stable) induced by OLR disturbance in an anaerobic digester used for treating FW. The results show that there was no clear correlation between ecological parameters and process stability. Most of the bacteria showed redundant functional adaptation to increased OLR. Therefore, new steady-state microbial community profiles were observed after disturbance. However, the genera *Syntrophomonas* and *Treponema* appeared to be resilient groups; their abundances were closely related to process deterioration. This observation provides insight into the key microbial groups that control the operation of anaerobic digesters. Moreover, the succession of methanogens during the disturbance phase was unsuited for the metabolic function needed at high OLR. This contradiction was the fundamental reason for the process deterioration. Thus, methanogenesis is the restricting step that impedes stable and efficient operation of digesters. Identifying and breeding high-efficiency methanogens will be helpful for breaking the technical bottleneck of process instability and achieving stable operation during high OLR. These findings improve the understanding of the correlation between microbial communities and process stability, and provide a theoretical basis for the efficient and stable operation of anaerobic digesters for treating FW.

## Methods

### Inoculum and substrate

The inoculum used in this study was obtained from a rural household biogas digester operated at ambient temperature. Its characteristics are as follows: pH 7.5 ± 0.3, total solids (TS) 9.1 ± 0.1 %, and volatile solids (VS) 5.4 ± 0.1 %. The FW was collected from a student dining facility at Chongqing University, Chongqing, China, and was shredded using a Robot-Coupe Shredder to less than 5 mm in diameter after removing bones, eggshells, napkins, plastic, and other indigestible materials. The prepared materials were stored at −18 °C in 4-L plastic storage bags. Before its use, the frozen feedstock was thawed at 4 °C for no more than 1 week. The FW had a pH of 6.4 ± 0.2 and TS and VS contents of 28.4 ± 0.7 and 26.5 ± 0.7 %, respectively.

### Reactor setup and operation

The lab-scale AD experiment was carried out in an automatic completely stirred tank reactor (CSTR) (BMR-A50U, Auzone, Shanghai, China) with a working volume of 30 L. The reactor was equipped with a mechanical agitator, and the rotary speed was set at a rate of 60 rpm, with a 1-h stirring and 2-h break repetitive sequence. A constant temperature of 36 ± 1.0 °C was maintained with a water jacket heated from a thermostat. Electrodes for continuous monitoring of temperature, pH, and redox potential were inserted into the digester in sealed sockets. Gas production and composition were also monitored on-line using an infrared detector.

Initially, the digester was filled with the above-mentioned inoculum (30 L). After pre-incubation for 2 weeks, the digester was initiated at an OLR of 3 g VS L^−1^ d^−1^ and operated in a daily fill and draw mode. Once the digester reached steady state conditions (determined by a constant methane yield and VS_r_), OLR disturbance was introduced. Thus, the experiment was divided into four periods: stable operation (Phase I, 0–45 days), OLR disturbance (Phase II, 46–90 days), recovery (Phase III, 91–120 days), and new stable operation (Phase IV, 121–150 days). The specific OLR at each stage was 3, 4–6 (increased with an interval of 1 g VS L^−1^ d^−1^ every 15 days), 0, and 1–3 g VS L^−1^ d^−1^ (increased with an interval of 1 g VS L^−1^ d^−1^ with a retention time for each OLR of 7, 7, and 16 days), respectively.

### Physico-chemical analysis

Except for the online monitored pH, gas production and composition, TS, VS, VFA, TA, individual VFAs and TAN were also measured. Specifically, the TS and VS were measured every 3 days according to standard methods, and the VS_r_ was calculated according to the equation introduced by Koch et al. [40]. TAN was analyzed every 3 days using a DR-2800 spectrophotometer (HACH, USA), and the FAN concentration was calculated based on the method described by Körner et al. [41]. VFA and TA were analyzed daily according to our previous report [4]. For the measurement of individual VFAs, samples were collected every 3 days and centrifuged immediately at 10,640×*g* for 10 min. Then, the supernatant was filtered through a 0.22-µm syringe filter before being acidified to pH 2.0 ~ 3.0 with formic acid. The prepared samples were analyzed using a Gas chromatograph (Agilent 7890A, USA) with a capillary column (DB-FFAP, Agilent) coupled to a hydrogen flame ionization detector (FID).

### Microbial analysis

To analyze the microbial community dynamics induced by OLR disturbance, 0.3 g of digestate operated under different stages (Day 45, 90, 120, and 150, respectively) was used for genomic DNA extraction using the E.Z.N.A Soil DNA kit (OMEGA, USA) following the manufacturer’s instructions. 16S rRNA genes segments were amplified from the obtained DNA using bar-coded primer pairs of 27F (5′-AGAGTTTGATCCTGGCTCAG-3′) and 533R (5′-TTACCGCGGCTGCTGGCAC-3′) for bacteria and 344F (5′-ACGGGGCTGCAGCAGGCGCGA-3′) and 915R (5′-GTGCTCCCCCGCCAATTCCT-3′) for archaea. The PCR amplification conditions for bacteria were as follows: heating at 95 °C for 2 min; 25 cycles of denaturing (95 °C; 30 s), annealing (55 °C; 30 s), and extension (72 °C; 1 min); and a final elongation (72 °C, 10 min). The amplification program for archaea was similar to that for bacteria except that the cycles of thermal cycling were 27 rather than 25.

The PCR products were sent to Shanghai Majorbio Bio-pharm Technology Co., Ltd (Shanghai, China) for sequencing on the Roche GS FLX 454 pyrosequencing platform to generate 400-bp sequence reads. Before sequencing, the amplified 16S rRNA gene was purified, quantified, and then mixed at equal concentrations. The raw nucleotide sequence reads were sorted, trimmed, qualified, and then clustered to OTUs following the procedures described by Yi et al. [42]. Chimeras were checked and removed from the data using UCHIME described by Edgar et al. [43].

Various ecological indices were applied, which mainly were based on the microbial resource management concept. Richness was determined as the total number of detected OTUs. The Jaccard index was computed as it measures the similarity between samples and hence can be used as an index for dynamic community changes. Additionally, we defined the Lorenz curve and the derived Gini coefficient for each sample, which are related to information about community organization [6, 18, 44]. The Gini coefficient describes a specific degree of evenness of a microbial community, and the higher the Gini coefficient, the more uneven is the community. Significant differences in richness and community organization among phases were analyzed using one-way analysis of variance (ANOVA) followed by Duncan’ s multiple range test (P < 0.05) with SPSS software (version 20).

For the taxonomy-based analysis, the SILVA database project (http://www.arb-silva.de) was used as a repository for aligned rRNA sequences. The final nucleotide sequences obtained have been deposited in NCBI under the accession number SRP065754.

## Abbreviations

AD: anaerobic digestion
FW: food waste
OLR: organic loading rate
VS_r_: volatile solids removal rate
VFA: volatile fatty acid
TA: total alkalinity
TAN: total ammonia–nitrogen
FAN: free ammonia
OTUs: operational taxonomic units
SAO: syntrophic acetate oxidation
HM: hydrogenotrophic methanogenesis
AM: aceticlastic methanogenesis
SAOB: syntrophic acetate oxidizing bacteria
EPS: exopolysaccharides
SMA: specific methanogenic activity
TS: total solids
VS: volatile solids
CSTR: completely stirred tank reactor
FID: flame ionization detector
